# Development of ssDNA Aptamers for Diagnosis and Inhibition of the Highly Pathogenic Avian Influenza Virus Subtype H5N1

**DOI:** 10.3390/biom10081116

**Published:** 2020-07-28

**Authors:** Sang-Heon Kim, Jae-Woo Choi, A-Ru Kim, Sang-Choon Lee, Moon-Young Yoon

**Affiliations:** 1 Department of Chemistry and Research Institute of Natural Sciences, Hanyang University, Seoul 133-791, Korea; konasi2@naver.com (S.-H.K.); kimr2122@gmail.com (A.-R.K.); 2 Center for Theragnosis, Biomedical Research Institute, Korea Institute of Science and Technology (KIST), Seongbuk-gu, Seoul 136-791, Korea; 091685@kist.re.kr; 3 Department of Chemistry, Georgia State University, Atlanta, GA 30303, USA; lsckhs@hanmail.net

**Keywords:** avian influenza, HPAI, H5N1, SELEX, aptamer, hemagglutination

## Abstract

Avian influenza (AI) has severely affected the poultry industry worldwide and has caused the deaths of millions of birds. Highly pathogenic avian influenza virus is characterized by high mortality and the ability to transmit from birds to humans. Early diagnosis is difficult because of the variation in pathogenicity and the genetic diversity between virus subtypes. Therefore, development of a sensitive and accurate diagnostic system is an urgent priority. We developed ssDNA aptamer probes to detect AI viruses. Through seven rounds of SELEX to search for a probe specific to the highly pathogenic AI virus subtype H5N1, we identified 16 binding aptamers and selected two with the highest binding frequency. These two aptamers had strong binding affinities and low detection limits. We found that they could bind more specifically to H5N1, as compared to other subtypes. Furthermore, these aptamers inhibited hemagglutination, which is caused by the virus surface protein hemagglutinin. Our results indicate that our screened aptamers are effective molecular probes for diagnosing H5N1 and can be used as therapeutic agents to inhibit viral surface proteins. Sensitive diagnosis and suppression of avian influenza will help maintain a stable and healthy livestock industry, as well as protect human health.

## 1. Introduction

Avian influenza is caused by the avian influenza A virus, which occurs naturally in wild aquatic birds. Infection in poultry can cause serious damage to the global poultry market because the virus is highly contagious and has high mortality rates [1,2]. Human infections with avian influenza viruses, though rare, have been reported sporadically. In 1997, human infections with the highly pathogenic avian influenza (HPAI) A virus, especially H5N1, were reported during an outbreak in poultry in Hong Kong, in China. Since 2003, H5N1 has spread from areas in Asia to other regions of the world and has become endemic in poultry populations in some countries. Outbreaks have resulted in numerous poultry infections and several human cases with high mortality [3,4,5]. Circulation of some avian influenza virus subtypes in poultry, such as A(H5) and A(H7), are of public-health concern because these viruses cause severe disease and have the potential to mutate and increase transmissibility in humans [6]. Although it is unclear whether the currently circulating avian influenza virus will lead to a pandemic, the diversity among the human-infectious avian influenza virus strains is of concern. Thus, a thorough investigation of every zoonotic infection in both animal and human populations and a pandemic-preparedness plan are needed [7]. Currently, avian influenza can only be diagnosed via detection of cultured avian influenza virus, viral RNA, or viral antigen in the specimen obtained from a bird suspected of infection. Additionally, a blood test is performed to confirm the increase in antibodies against the virus [8,9,10]. This method is time-consuming and costly. It is also not suitable for field diagnostics, because an accurate subtype cannot be determined, due to the genetic diversity of avian influenza virus subtypes [11]. Therefore, a new diagnostic system is required that can quickly and accurately identify avian influenza virus subtypes. Additionally, existing antiviral drugs to treat avian influenza virus infection have limited efficacy because of viral resistance. Most widely used antiviral medications are neuraminidase inhibitors such as oseltamivir and zanamivir. However, oseltamivir-resistant viruses have been reported these days [12,13]. Therefore, it is necessary to develop an antiviral agent with a mechanism of action that is different from conventional ones.

A DNA aptamer is a short oligonucleotide that binds to a specific target with strong affinity. Due to its single stranded structure, it can fold into specific structure (secondary, tertiary). Aptamer binding is mediated by a combination of several weak interactions, such as hyderogen bond, electrostatic interaction, van der Waals forces, and π-π stacking. Based on aptamer’s specific structure and binding forces, it can bind to various targets, from a small metal ion to a large cell. The ssDNA (single-stranded DNA) aptamer has several advantages for early diagnosis, especially with regard to point-of-care diagnostics. Aptamers have a small molecular weight and high stability compared to antibodies. They are also easy to modify and inexpensive to produce at a mass scale. Therefore, they can be used as capture probes as a substitute for antibodies [14,15,16]. An aptamer is selected as a best bind to a target from approximately 10^15^ random library sequences [17,18,19], via a screening process called SELEX. There are many SELEX methods, and the most specific and high-binding-affinity aptamers are screened through sequential binding, washing, elution, and amplification [20,21]. We found our aptamers by using a membrane-filtration spin column process, where the target virus was too large to pass through the membrane, while the oligonucleotides, which were smaller, were able to pass through. Thus, the unbound aptamers were separated by centrifugation [22].

In this study, we performed seven rounds of SELEX and found two aptamers that specifically bind to the H5N1 virus. One aptamer was also found to inhibit viral activity effectively, for which we anticipate could make it a potential therapeutic agent.

## 2. Materials and Methods

### 2.1. Materials

Template (containing 30 random bases), primers, and all oligonucleotides were synthesized by Bioneer (Daejeon, Korea). Pfu DNA polymerase was purchased from Biofact (Daejeon, Korea), and restriction enzymes and DNA ligases were purchased from Takara Bio (Shiga, Japan). Chicken red-blood cells (RBCs), packed at 5%, were purchased from Innovative Research (Detroit, MI, USA). Allantoic fluid and influenza A virus subtypes (H5N1 (A/wild duck/Korea/SNU50-5/2011), H1N1 (A/Puerto Rico/8/1934), H9N2 (A/Duck/Hong Kong/702/1979), recombinatnt H5N1(*p*), and recombinant H5N6) were provided by the Seoul National University, College of Veterinary Medicine. M13 phage was purchased from New England Biolabs (Ipswich, MA, USA). Reduced graphene oxide (rGO) was purchased from Graphene Supermarket (Ipswich, MA, USA).

### 2.2. Preparation of Random ssDNA Library

To prepare the ssDNA library pool of 30 mer random sequences (5′-ATGCGGATCCCGCGC-(N30)-GCGCGAAGCTTGCGC-3′), asymmetric polymerase chain reaction (PCR) was performed with a forward primer, 5′-ATGCGGATCCCGCGC-3′ (including a BamHI site), and a reverse primer, 5′-GCGCAAGCTTCGCGC-3′ (including a HindIII site). Pfu polymerase was used for amplification. Template DNA was amplified by asymmetric PCR, using 100 μM forward and 10 μM reverse primers to obtain a single-stranded DNA. PCR was performed with the following conditions: denaturation at 95 °C for 3 min, incubation at 95 °C for 40 s, annealing at 55 °C for 40 s, and extension at 72 °C for 20 s. The last three steps were repeated for 35 cycles. Finally, an additional extension step was carried out at 72 °C for 10 min. After PCR, amplified ssDNA was extracted from a native gel by the crush-and-soak method [23]. The PCR product was electrophoresed on a 12% native gel to separate double-stranded (ds) and ssDNA. After staining DNA with ethidium bromide (EtBr), we cut out the gel where a single-stranded DNA band was shown and was crushed into the crush-and-soak buffer (500 mM ammonium acetate, 0.1 mM ethylenediaminetetraacetic acid, and 0.1% SDS). The mixture was rotated overnight at room temperature. Then, 0.5 vol of 3M Sodium acetate and 3.5 vol of 100% ethanol solution were added, and the mixture was kept at −20 °C, overnight, to purify the ssDNA [24]. Precipitates were later separated by centrifugation (14,000 rpm, 15 min). The solution was dried at 65 °C in a dry oven and dissolved with 50 µL of water. ssDNA concentration was measured with a UV/vis spectrophotometer and was then used for screening.

### 2.3. SELEX Using the Membrane Filtration Column Method

SELEX was performed with 30 kDa cutoff Vivaspin ultrafiltration spin columns (Sartorius Stedim Biotech GmbH, Göttingen, Germany) (Supplementary Materials Figure S1), which is a method used for large-sized targets, such as the H5N1 virus (80–120 nm). First the H5N1 virus was dissolved in Tris buffer (pH 7.4) containing 1 μg/mL ssDNA. To remove any weakly bound or unbound aptamers, membrane filtration, using the 30 kDa cutoff, followed by separation by centrifugation at 14,000 rpm for 20 min were performed. Next, eluted virus-bound ssDNA was amplified by using asymmetric PCR and was checked with 12% native polyacrylamide gel electrophoresis (PAGE). The ssDNA in the native PAGE gel was further purified by gel elution and the crush-and-soak method. To select aptamers with a high affinity and specificity, seven rounds of SELEX were performed, using harsh conditions such as increasing salt and detergent concentrations and decreasing binding times and target concentrations (Rounds 1, 2: 10 HAU; Rounds 3, 4: 5 HAU; Rounds 6, 7: 2 HAU) (Supplementary Table S1). To increase the specificity of ssDNA toward the target, a negative round (fifth round) of SELEX was performed with bovine serum albumin (BSA), and the BSA-unbound ssDNA was eluted by centrifugation.

### 2.4. Analysis of Aptamer Sequences and Secondary Structures

Selected ssDNA obtained from the final round of the SELEX process was amplified by symmetric PCR to generate dsDNA needed to clone the specific H5N1-binding aptamer. Amplified dsDNA was digested using restriction enzymes (Hind III and Bam HI) and was ligated into the pET28a(+) expression vector. The pET28a-dsDNA (ligation product) was checked with 0.8% agarose gel electrophoresis, and the product sequence was analyzed by Macrogen (Seoul, Korea). Opensource Mfold software was used to predict aptamer secondary structures [25,26].

### 2.5. Optimization of the Reduced Graphene Oxide (rGO)-Based Aptamer Characterization Test

Aptamers with FAM (6-carboxyfluorescein) labeling at the 5′-end were synthesized. Prior to characterization, the fluorescence-quenching conditions of the FAM-labeled aptamers on rGO were optimized. rGO can adsorb ssDNA through electrostatic and π−π stacking interactions [27]. Furthermore, rGO was able to adsorb the fluorescent molecules and quench the fluorescence signal, which could be applied to a sensitive detection system. Fluorescence at the 5′-end of the aptamer was quenched in the presence of rGO in a concentration, incubation, and time-dependent manner.

### 2.6. Aptamer Binding Assay by Fluorescence Recovery Analysis

The binding affinities of H5N1 influenza virus binding aptamer 1 (HBA1) and HBA2 were estimated via fluorescence recovery signal from the rGO surface. Each concentration of HBA1 and HBA2 (0, 0.078, 0.016, 0.031, 0.063, 0.13, 0.25, and 0.5 μM) was incubated for 60 min with 0.25 μg/μL of rGO, to quench the fluorescence signal. Next, 0.8 HAU of H5N1 virus was added, and the fluorescence intensity was measured after 30 min of centrifugation at 14,000 rpm, to determine the dissociation constant (Kd). Kd was determined by Hill equation. Fluorescence recovery of truncated HBAs (tHBAs) was also estimated as described above.

### 2.7. Aptamer Specificity Test Against Virus Subtypes

Specificity testing against other avian influenza virus subtypes (H1N1, H5N1, H5N1(*p*), H5N6, and H9N2) and non-influenza virus (M13 bacteriophage) was performed. We also tested PBS as a control. HBA1 (0.25 μM) was incubated with 0.25 μg/μL of rGO for 60 min. All viruses were dilluted by using allantoic fluids. Then, 0.8 HAU of viruses (H1N1, H5N1, H5N1(*p*), H5N6, and H9N2) and 10^11^ pfu of M13 bacteriophage were added to each reaction tube. After centrifugation, the supernatant was removed, and the fluorescence intensity was measured by using a fluorimeter. The signal due to allantoic fluids was subtracted from all virus signals, to determine exact intensity.

### 2.8. Estimation of the Detection Limit

The limit of detection (LOD) of the H5N1 virus was also measured. HBA1 (0.25 μM) was incubated with 0.25 μg/μL of rGO for 60 min. Next, 0, 0.0125, 0.025, 0.05, 0.1, 0.2, 0.4, and 0.8 HAU of H5N1 virus was added to detach ssDNA from the rGO surface. Fluorescence intensity of the supernatant was measured after centrifugation. The LOD value was calculated from Formula (1), as follows, provided by the International Union of Pure and Applied Chemistry (IUPAC):

LOD = 3*SD/slope(1)

### 2.9. Hemagglutination Assay

The concentration of hemagglutination antigen (HA) of each virus (H1N1, H5N1, and H9N2) was determined via HA assay, wherein the outcome was the reciprocal of the last dilution of influenza virus antigen that can still generate complete hemagglutination. Viral antigen was two-fold serially diluted (1:2^0^ to 1:2^10^) in 50 μL of PBS in a round-bottomed 96-well plate. Dilutions of allantoic fluid were used as controls. Then, 50 μL of 0.5% chicken RBCs was then added to the wells, to make a total of 100 μL and incubated with the viral mixture, at room temperature, for 40 min. Agglutination patterns were then read, and the unit of HA was determined [28].

### 2.10. Hemagglutination Inhibition Assay

A hemagglutination inhibition assay was conducted to evaluate the the ability of the ssDNA aptamer to inhibit hemagglutination caused by the H5N1 virus. The assay was conducted in round-bottomed 96-well plates. PBS buffer (50 μL, pH 7.4), including 10 μL of H5N1 virus at 16 HAU, and aptamers at varying concentrations (2500 nM) were mixed, added to each well, and incubated with the viral mixture, at room temperature, for 40 min, before 50 μL of 0.5% chicken RBCs was added to each well, to make total 100 μL. All agglutination reactions were incubated for 30 min before being photographed. PBS was used as a negative control. Similar hemagglutination tests were also performed on other virus subtypes (H1N1 and H9N2).

### 2.11. Statistical Analyses

We used OriginPro for all statistical analyses. All mean values and standard deviations were estimated from triplicate test results.

## 3. Results

### 3.1. Identification and Structural Analysis of H5N1-Binding Aptamers (HBAs)

We confirmed the presence of smear bands of single-stranded and linear double-stranded DNA with other dispersed bands after 12% DNA native gel electrophoresis. (Supplementary Figure S2A). We extracted only the ssDNA at the 30 bp marker via the crush-and-soak method and gathered it. After extraction, the purified ssDNA was checked again for presence at the 30 bp marker on a 2.5% agarose gel (Supplementary Figure S2B). Through seven rounds of the SELEX process, we identified 16 aptamer sequences and selected two of them (HBA1 and HBA2) based on their high binding frequency (Table 1). The secondary structures of the aptamers were analyzed, using the Mfold program, and then we truncated the aptamer candidates by structure (Supplementary Figure S3).

### 3.2. Characterization of H5N1-Binding Aptamers

Binding conditions were optimized prior to characterization. Then, rGO at 0.25 μg/μL was found to completely quench the fluorescence signal, and this condition was used to develop an H5N1-specific aptamer. A 60 min incubation time was determined to be optimal for rGO and aptamers (Supplementary Figure S4).

**Figure 1:**
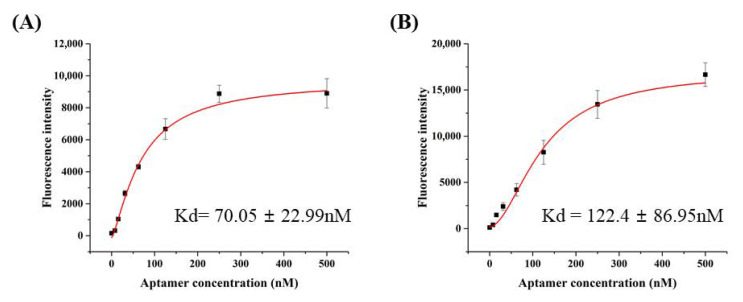
Characterization of aptamers via fluorescence quenching and recovery assay. (**A**) Binding affinity of HBA, Kd = 70.05 nM. (**B**) Binding affinity of HBA2, Kd = 122.4 nM.

**Figure 2:**
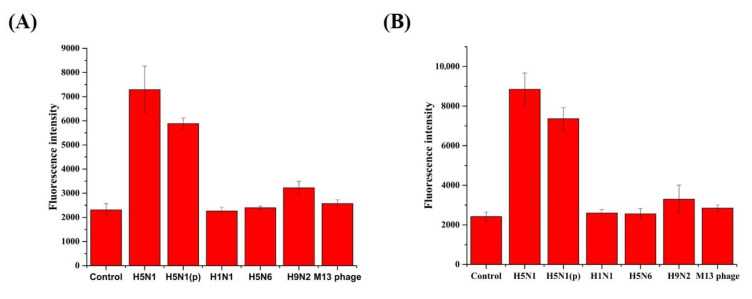
Specificity test for the selected aptamers. Each aptamer (HBA1 (**A**) and HBA2 (**B**)) specifically bound to H5N1 and H5N1(*p*), which was significantly greater specificity than for other virus subtypes (H1N1, H5N6, and H9N2) and non-influenza virus (M13 phage).

HBA1 and HBA2 properties were analyzed by using the fluorescence recovery signal based on the above-optimized quenching conditions. The binding affinities of the HBAs are shown in Figure 1. Recovered fluorescence intensity increased with aptamer concentration, which reached saturation near 0.25 μM of the aptamer. The Kd for the HBAs was 70 nM (HBA1) and 122 nM (HBA2). In Supplementary Figure S5, Kd for the tHBAs was 491 nM (tHBA1) and 1323 nM (tHBA2). Both H5N1 binding aptamers showed significantly higher fluorescence recovery against the two different H5N1 virus subtypes than against H1N1, H5N6, H9N2, or non-avian influenza virus (M13 phage) (Figure 2).

### 3.3. Detection Limit for HBA1 and HBA2

**Figure 3:**
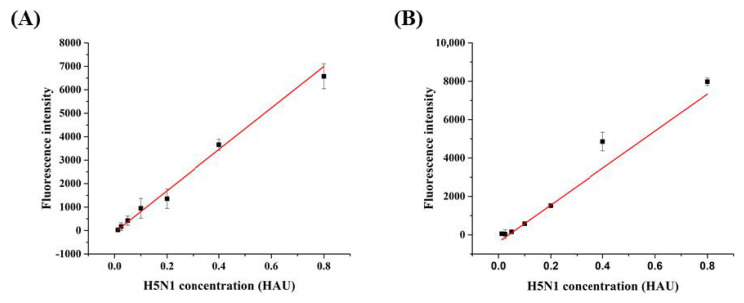
Limit of detection (LOD) for H5N1: (**A**) HBA1 LOD = 0.08 HAU, and (**B**) HBA2 LOD = 0.1 HAU.

The detection limits of HBA1 and HBA2 were determined via H5N1 concentration-dependent fluorescence recovery. Fluorescence intensity recovered, along with an increase in H5N1 virus concentration. The detection curve was saturated at a high H5N1 concentration, and testing in the low range of H5N1 concentration was performed, to acquire a linear range of detection. According to the recovery signal, a linear graph for H5N1 was obtained in the range of 0–0.8 HAU, and the LOD for HBA1 and HBA2 was determined to be 0.08 and 0.1 HAU, respectively (Figure 3).

### 3.4. Hemagglutination Inhibition Assay

**Figure 4:**
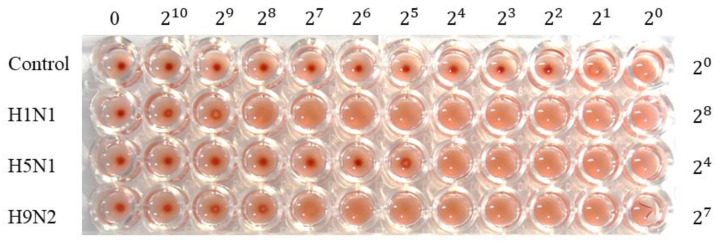
Hemagglutination assay for influenza virus. HA titers were H1N1 = 256 HAU, H5N1 = 16 HAU, and H9N2 = 128 HAU.

**Figure 5:**
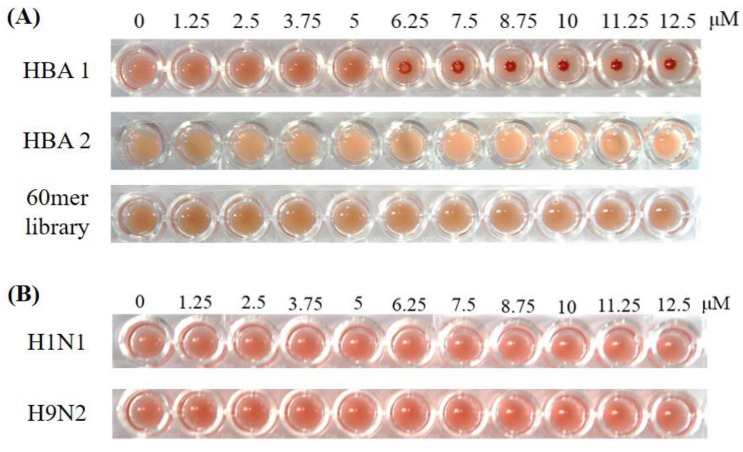
Hemagglutination inhibition assay for influenza virus with developed aptamers. (**A**) HBA1 showed hemagglutination inhibition of H5N1 at the lowest concentration, 6.25 μM. HBA2 showed a weak inhibition effect on hemagglutination up to a concentration of 11.25 μM. However, the 60 mer library that was nonspecific for H5N1 had no inhibitory effect. (**B**) HBA1 had no inhibitory effect on the other virus subtype.

RBCs agglutinated in the presence of all influenza virus subtypes, resulting in a lattice structure. Allantoic fluid dilutions were used as controls, and the HA unit was determined for each virus: control = 1 HAU, H1N1 = 256 HAU, H5N1 = 16 HAU, and H9N2 = 128 HAU (Figure 4). Figure 5A shows a top-down view of the hemagglutination reaction mixtures in round-bottomed microwell plates after the addition of aptamers. When the HBAs were added to the hemagglutination mixture, hemagglutination gradually diminished as HBA concentration increased. Clear agglutination was observed in wells containing 0, 1.25, 2.5, 3.75, and 5 μM of HBA1, and partial agglutination was observed in wells containing 6.25, 7.5, 8.75, 10, 11.25, and 12.5 μM of HBA1. When HBA1 concentration was >6.25 μM, a button of RBCs was observed at the bottom of the microtiter well. With the HBA2 aptamer, a weak inhibition effect was present at concentrations >10 μM. The faint red O-ring-shaped dot was found on the RBC mixtures. In contrast, the hemagglutination reaction was observed at all concentrations when the 60 mer library was used (Figure 5A). The inhibition test using tHBAs showed weak hemagglutination inhibition at 47 μM (Supplementary Figure S6). In the presence of other virus types (e.g., H1N1 and H9N2), HBA1 had no inhibitory effect, even at 12.5 μM (Figure 5B).

## 4. Discussion

Prior to the screening process, ssDNA was amplified via asymmetric PCR. Because the amplified 60 mer libray was single-stranded DNA, it was observed at the 30 bp marker as smear bands (Supplemntary Figure S2A). Other bands were the common by-products generated in the PCR process and were removed during the purification step. We retrieved the ssDNA at the 30 bp marker only after elimination of the other PCR products. The amplified ssDNA library was used for screening.

Typically, many SELEX processes contain steps for target immobilization to solid supports [29]. However, this can introduce nonspecific binding to the aptamer, limiting the enrichment of most specific aptamers during the SELEX process [30]. Many simple isolation methods for bound aptamers in supernatants have been used generally in recent studies [31,32]. Herein, we got 16 different sequences through seven rounds of the SELEX process, using spin columns. Unbound ssDNA was eliminated after passing through the membrane, but the ssDNA bound to the target remains in the column. Among the identified aptamers, HBA1 and HBA2 were selected for their superior target-binding frequency (Table 1). As shown in Supplementary Figure S3, we selected the structures with the lowest Gibbs free energy (ΔG) among the predicted structures. The selected structures had a unique stem-and-loop structure, which is known to be important for binding [33]. Thus, HBA1 and HBA2 were truncated without structure transformation, to determine the contribution of each stem–loop region.

Reduced graphene oxide (rGO) was used to characterize HBAs, and rGO can absorb ssDNA due to the π-π stacking interaction between graphene and nucleic acid. Furthermore, when the green fluorescent is near the rGO, it is quenched due to the fluorescence resonance energy transfer effect [34]. When HBA1 and HBA2 were adsorbed on the rGO surface, the fluorescence intensity decreased inversely with rGO concentration. The optimized binding conditions indicate strong adsorption between rGO and ssDNA within a short time during simple incubation (Supplementary Figure S4).

Aptamer binding characters are estimated through the recovery of quenched fluorescence (Figure 1). When the absorbed aptamer meets the target virus, the aptamer undergoes a conformational change, which weakens the interaction between graphene and nucleic acid [35]. In this study, the absorbed aptamer detached from rGO when it bonded with H5N1, and the quenched fluorescence signal was also recovered. The binding affinity on the nanomolar range showed that HBA1 and HBA2 had very strong affinities to H5N1. The fluorescence recovery test, using truncated HBAs (tHBAs), showed much lower binding compared with the whole aptamers (Supplementary Figure S5). None of the separated stem-and-loop structures in the HBAs worked effectively alone compared with the full-length structure, indicating that stem-and-loop structures work cooperatively.

Moreover, we compared fluorescence recovery by using another H5N1 strain, three differnet virus subtypes, and non-avian influenza virus for specificity test (Figure 2). Interestingly it showed quite different signals among each virus. Much higher fluorescnece recovery of two different strains of H5N1 means that our aptamers bind very specifically to the H5N1. Even at the excess concentration of M13, bacteriophage showed almost non-binding signal, thus demonstrating that our aptamers can distinguish H5N1 with very high specificity via a simple one-step test. Therefore, these novel H5N1-specific aptamers (HBA1 and HBA2) could be applied to point-of-care diagnostic systems for livestock.

The very low detection limits of the HBAs suggest the probability of a diagnostic sensor system for early H5N1 detection (Figure 3). The existing two avian influenza virus diagnostic methods are PCR and ELISA, and most avian influenza has been confirmed through detecting virus directly or testing serum for observing immune response to the virus [36]. Many studies have shown that aptamers can be good alternatives to existing diagnostic methods in food safety [37]. Our HBA-based detection system is rapid and cost-effective compared to other methods. These results further underscore the potential for HBAs in point-of-care diagnostics.

**Figure 6:**
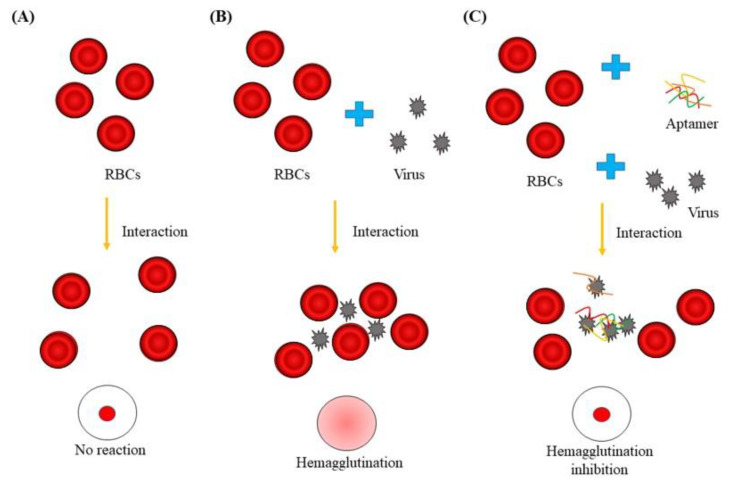
Schematic of the hemagglutination inhibition assay. (**A**) RBCs do not bind together and will sink to the bottom of the well plate. This is visible as a red dot at the center of the well. (**B**) A number of viruses have hemagglutinins that cause agglutination of RBCs, resulting in hemagglutination and formation of a lattice structure that results in a red color throughout the well. (**C**) Virus-specific aptamer, the virus, and the RBCs were added to the well plate. The virus-specific aptamer inhibits agglutination, which is visible as a red dot at the bottom of the well.

We also explored how the aptamers bind and affect the H5N1 virus. Some viruses, including AI virus, attach to receptor proteins on RBC surfaces, resulting in hemagglutination. The agglutinated RBCs were suspended and appeared as a diffused reddish solution. However, when HA protein binding to sialic-acid receptors was blocked by the aptamer inhibitors, no hemagglutination occurred, and the RBCs were not constrained by the lattice; the RBCs consequently settled at the bottom of the well (Figure 6) [38]. Prior to the inhibition assay, we performed a hemagglutination assay, to estimate the HA titer for each virus. In the results, each virus showed its biological activities forming hemagglutination (Figure 4).

To verify the inhibition ability of each selected aptamer against the virus binding to RBCs, we performed a hemagglutination inhibition assay with the minimum concentration of virus required to aggregate RBCs. A clear red dot at the RBC mixtures means that HBA1 effectively inhibits the virus binding to RBCs. HBA2 also showed an ability to inhibit hemagglutination, though not as strong as HBA1 (Figure 5A). In contrast to screened aptamer, the scramble 60 mer library hardly inhibits the virus binding, indicating that only specific aptamers exhibited an inhibitory effect. Likewise, a weak hemagglutination inhibition, even in using a much higher concentration, means that truncated HBAs did not bind to the virus well, and it was consistent with our binding assay results (Supplementary Figure S6). We also performed an inhibition test, using other virus subtypes, to evaluate aptamer specificity, which led to different results from those gathered from the H5N1 subtype. No inhibitory effect of the same aptamers in the presence of H1N1 and H9N2 demonstrated that our HBAs, especially HBA1, specifically bonded to the HA of the H5N1 virus and inhibited it (Figure 5B). Nowadays, anti-influenza drugs are mostly M2 ion channel inhibitors and neuraminidase inhibitors. Hemagglutinin also plays a critical role in the viral binding and entry process. Hemagglutinin is becoming a promising target for developing avian influenza virus inhibitors [39]. There are many studies about the correlation between hemagglutination inhibition and viral neutralization [40,41]. In this work, our developed aptamer interrupted the hemagglutinating ability of the AI virus and might be further developed, specifically to interfere with virus/receptor-binding, and that this could open new perspectives for antiviral approaches, which would have to be investigated in the future.

## 5. Conclusions

We developed H5N1-specific aptamers by using a seven-round membrane-filtration SELEX method. The selected aptamers showed a unique stem-and-loop structure. We investigated the binding characters of the aptamers by using rGO and determined Kd = 70–120 nM and LOD = 0.08–0.1 HAU. Additionally, these aptamers preferentially bonded to H5N1, making them sufficient for use as detection probes for the avian influenza virus H5N1. Furthermore, this simple and cheap diagnostic method can be beneficial for a point-of-care system, which can diagnosis avian influenza by using field samples. Consequently, HBA1 is expected to bind specifically to the AI virus and inhibit the virus’s biological activity, thus effectively inhibiting the hemagglutinin–glycan interaction between H5N1 and RBCs. In conclusion, HBA1 and HBA2 can be used to diagnose avian influenza in place of traditional diagnostic methods, because they can quickly and accurately distinguish between the avian influenza subtypes.

## Figures and Tables

**Table 1:** Selected aptamer sequences after seven rounds of SELEX.

No.	F-Random Sequence-R	Frequency
1	GCGACCGTGTCAGCGGGGACTAGCGGTGTA	13/41
2	GGGTCTGAGGAGTGCGCGGTGCCAGTGAGT	9/41
3	GGGGGCTTGGACCGAGCGGTGTACGGCGCG	3/41
4	CGATCCACGAGAGTGCGCCCTGCATCCGAC	2/41
5	CGACGGCAGAGAGGCGCGGCGTCCCTTCGGTCC	2/41
6	GCCGTCAAGGGGTCAGTCACGGAAGCAAAG	2/41
7	GGTGTGGTTACACAGCCCGGACCGCCATGC	1/41
8	TCCCGGAGCAGCGGCAGCGTCCGGCTCT	1/41
9	GCATCGCAGTCACGCATGCGGAGTACGCCT	1/41
10	CCAGTGGATGGGGCGTGGGTTAGCTGCCGGAGG	1/41
11	TAGTCAGCAAGGTTCGCGGACCGGCGGGGT	1/41
12	GACGAAACGGAGGTGCGGCCCCTGCTGCC	1/41
13	GCGACCGTGTCAGCGGGGACTAGCGGTGTA	1/41
14	ACGTGAGCTTGTGCTGGACCTTGGCCACCC	1/41
15	GATGGGCGGCGTTCGCAGGGATCTGGCTGT	1/41
16	CAGGCACGACGTCGGGTCATCTGCAGCTCG	1/41
F(5′-ATGCGGATCCCGCGC-3′) R(5′-GCGCGAAGCTTGCGC-3′)		

## Supplementary Materials

The following are available online at https://www.mdpi.com/2218-273X/10/8/1116/s1. Figure S1: Schematic of SELEX method. Figure S2: Construction of ssDNA library. Figure S3: Secondary structure of aptamers. Figure S4: Optimization of the rGO concentration and reaction time. Figure S5: Characterization of truncated aptamers with the fluorescence quenching and recovery assay. Figure S6: Hemagglutination inhibition assay of influenza virus with truncated aptamers. Table S1: Experimental conditions of the SELEX.
