# Supplementary material for: Development of ssDNA Aptamers for Diagnosis and Inhibition of the Highly Pathogenic Avian Influenza Virus Subtype H5N1
Source: Biomolecules. 2020 Jul 28;10(8):1116. doi: 10.3390/biom10081116 (PMC7465229; doi:10.3390/biom10081116)
Supplement: Supplementary file 1 [file biomolecules-10-01116-s001.pdf]

Supplementary material

Supplementary figures

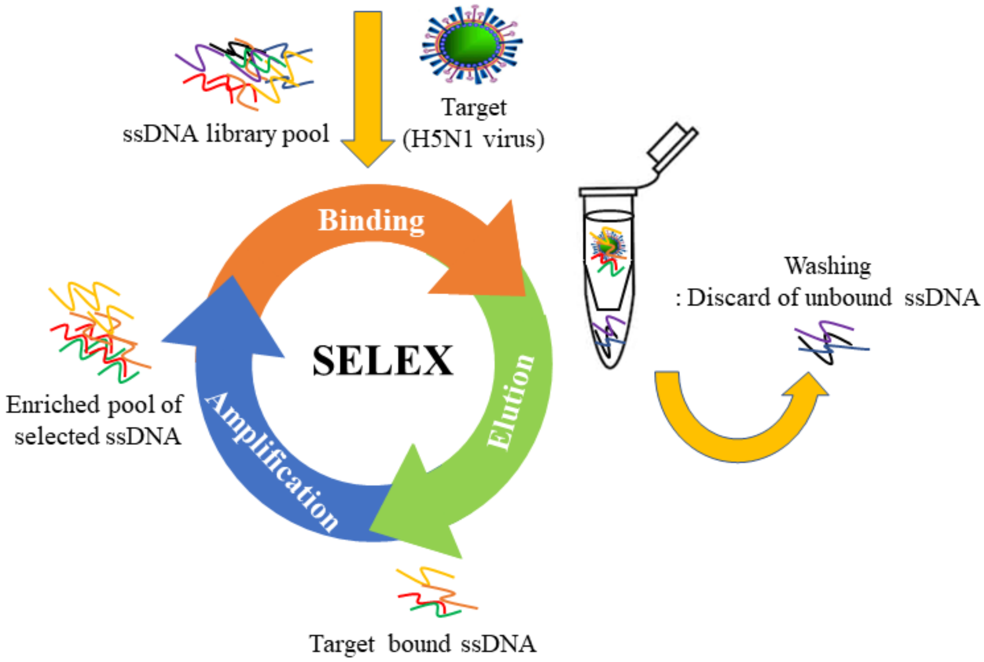

Figure S1. Schematic of SELEX method

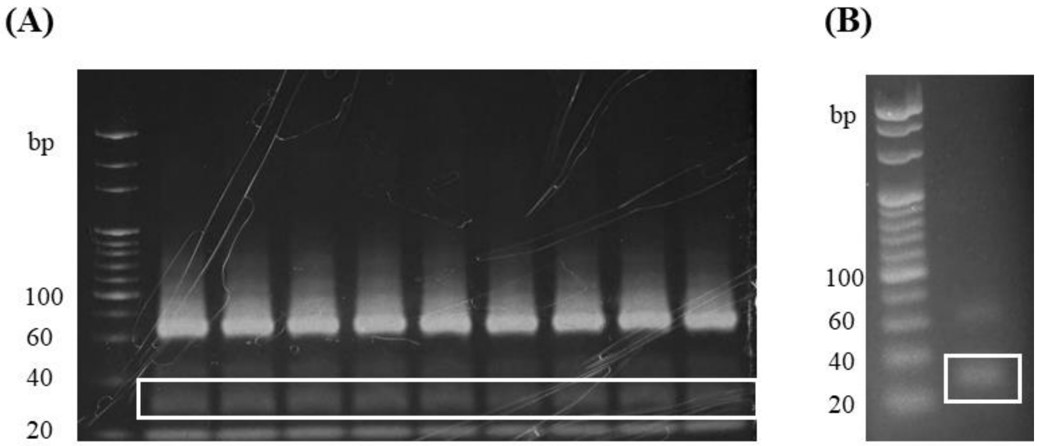

Figure S2. Construction of ssDNA library. (A) Identification of ssDNA in 12 % DNA native gel. ssDNA is shown by a white box. (B) Purified random ssDNA in whole PCR product (shown by a white box). M, 20bp DNA marker.

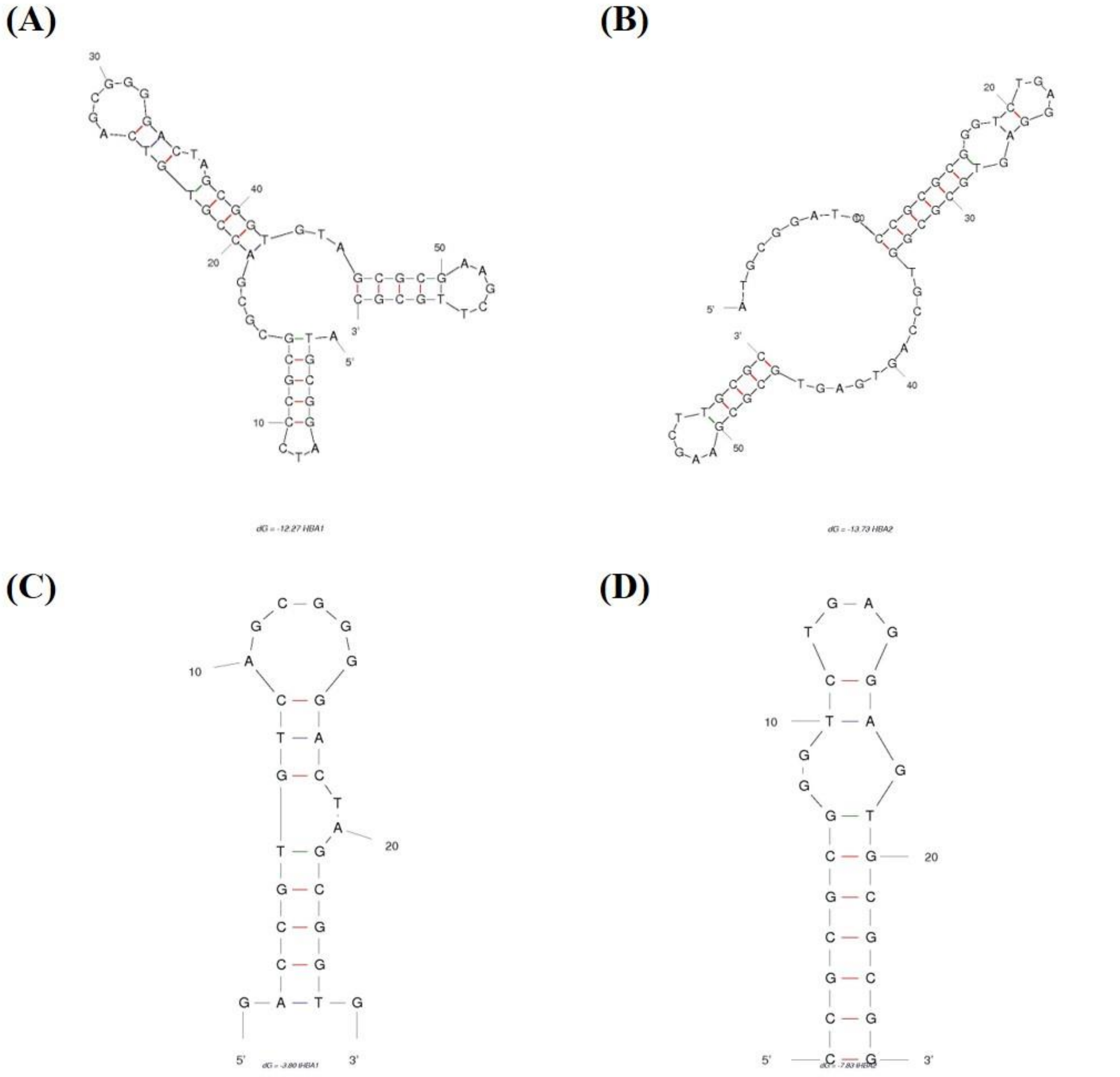

Figure S3. Secondary structure of aptamers. (A) HBA1 (ΔG = -12.27 kJ/mol), (B) HBA2 (ΔG= -13.73 kJ/mol) (C) tHBA1 (ΔG = -3.8 kJ/mol) (D) tHBA2 (ΔG = -7.83 kJ/mol) predicted using the M-fold software.

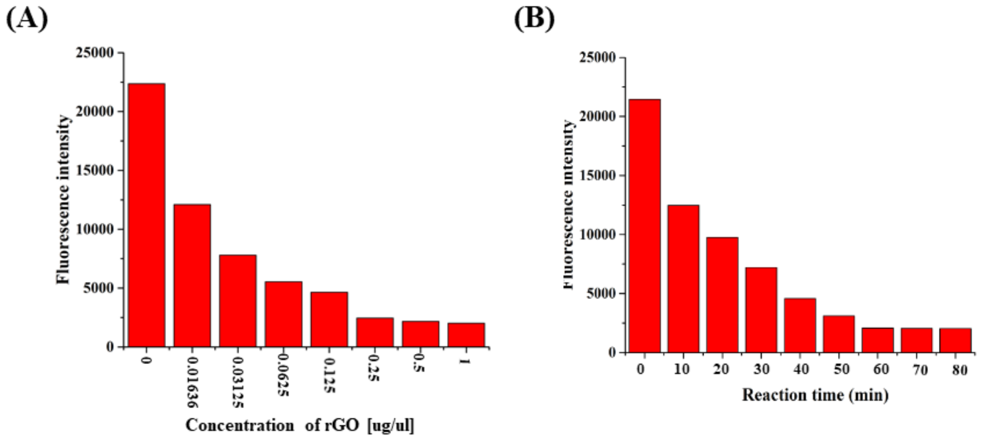

Figure S4. Optimization of the rGO concentration and reaction time. (A) Fluorescence intensity with a higher concentration of rGO. Maximum fluorescence was quenched with 0.25 ug/uL of rGO. (B) Fluorescence intensity quenched through reaction time. Fluorescent was quenched within 60 minutes.

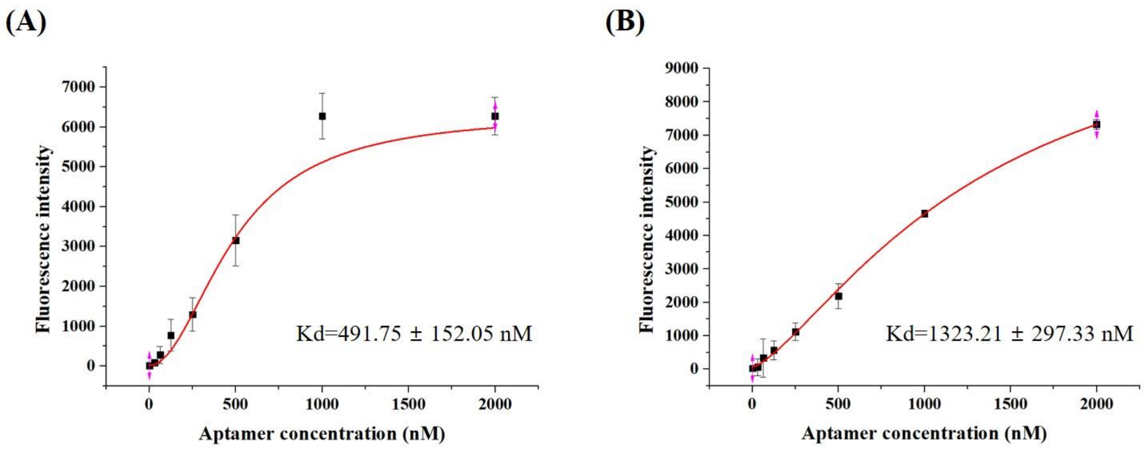

Figure S5. Characterization of truncated aptamers with the fluorescence quenching and recovery assay. (A) The binding affinity of tHBA1. K_d was determined to be 491.75 nM. (B) The binding affinity of tHBA2. K_d was determined to be 1323.21 nM.

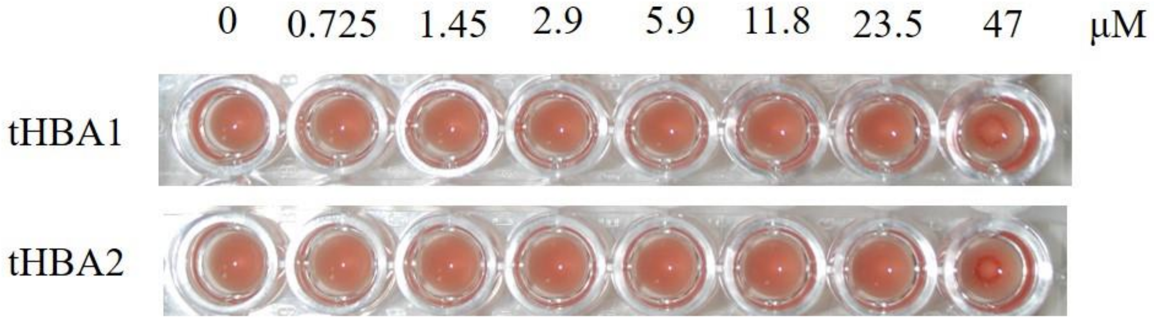

Figure S6. Hemagglutination inhibition assay of influenza virus with truncated aptamers (A) tHBA1 showed week inhibition of H5N1 at the lowest concentration of 47 μM. HBA2 showed a weak inhibition effect on hemagglutination at the concentration of 47 μM.

Supplementary Table

Table S1. Experimental conditions of the SELEX

Round	Buffer	Target	Time
1	20mM Tris	10 HAU	60 min
2	20mM Tris	10 HAU	60 min
3	20mM Tris, 50mM NaCl	5 HAU	45 min
4	20mM Tris, 50mM NaCl	5 HAU	45 min
5	20mM Tris	BSA (negative round)	30 min
6	20mM Tris, 100mM NaCl	2HAU	15 min
7	20mM Tris, 100mM NaCl	2HAU	15 min
